# Revisiting the genome-wide significance threshold for common variant GWAS

**DOI:** 10.1093/g3journal/jkaa056

**Published:** 2021-01-11

**Authors:** Zhongsheng Chen, Michael Boehnke, Xiaoquan Wen, Bhramar Mukherjee

**Affiliations:** Department of Biostatistics and Center for Statistical Genetics, University of Michigan School of Public Health, Ann Arbor, MI 48109-2029, USA

**Keywords:** multiple testing, FDR, family-wise error rate, Bonferroni correction, Benjamini–Hochberg, Bayesian false discovery probability

## Abstract

Over the last decade, GWAS meta-analyses have used a strict *P*-value threshold of 5 × 10^−8^ to classify associations as significant. Here, we use our current understanding of frequently studied traits including lipid levels, height, and BMI to revisit this genome-wide significance threshold. We compare the performance of studies using the *P *=* *5 × 10^−8^ threshold in terms of true and false positive rate to other multiple testing strategies: (1) less stringent *P*-value thresholds, (2) controlling the FDR with the Benjamini–Hochberg and Benjamini–Yekutieli procedure, and (3) controlling the Bayesian FDR with posterior probabilities. We applied these procedures to re-analyze results from the Global Lipids and GIANT GWAS meta-analysis consortia and supported them with extensive simulation that mimics the empirical data. We observe in simulated studies with sample sizes ∼20,000 and >120,000 that relaxing the *P*-value threshold to 5 × 10^−7^ increased discovery at the cost of 18% and 8% of additional loci being false positive results, respectively. FDR and Bayesian FDR are well controlled for both sample sizes with a few exceptions that disappear under a less stringent definition of true positives and the two approaches yield similar results. Our work quantifies the value of using a relaxed *P*-value threshold in large studies to increase their true positive discovery but also show the excess false positive rates due to such actions in modest-sized studies. These results may guide investigators considering different thresholds in replication studies and downstream work such as gene-set enrichment or pathway analysis. Finally, we demonstrate the viability of FDR-controlling procedures in GWAS.

## Introduction

There has been recent discussion in the statistical community on changing the standard *P*-value significance threshold for a single test from 0.05 to 0.005 (Benjamin *et al.* 2018; Amrhein *et al.* 2019; Wasserstein *et al.* 2019). Although the authors of the corresponding paper *(*Benjamin *et al.* 2018) commended human geneticists for using very stringent *P*-value thresholds to help ensure reproducibility, the cost of this strategy in current genetic studies is that many true genetic signals are not identified. The benefit is, of course, rigorous control of false positives.

To account for multiple testing in genome-wide association studies (GWAS), a fixed *P*-value threshold of 5 × 10^−8^ is widely used to identify association between a common genetic variant and a trait of interest. Risch and Merikangas (1996) suggested this strict *P*-value threshold for studying the genetics of complex diseases due to the many false positive discoveries reported by candidate gene studies at that time. Later, the International HapMap Consortium (Altshuler and Donnelly 2005), Dudbridge and Gusnanto (2008), and Pe’er *et al.* (2008) independently suggested near-identical thresholds for common variant (minor allele frequency [MAF] >5%) GWAS. Each group of investigators sought to control the family-wise error rate (FWER) through Bonferroni correction for the effective number of independent tests given the linkage disequilibrium (LD) structure of the genome; they used different approaches to estimate the effective number of independent tests. Based on these studies and reinforced by widespread use, the *P *=* *5 × 10^−8^ threshold soon became standard for common variant GWAS. Using this threshold has been remarkably successful in limiting false positive association findings, leading to robust and reproducible results in a field that prior to GWAS had reported many nonreplicable results.

Since the acceptance of the *P *=* *5 × 10^−8^ threshold a decade ago, there have been substantial experimental and methodological advances that have allowed study of many more common variants in much larger samples. The construction of denser genotype arrays (Burdick *et al.* 2006), development of genotype imputation (Li *et al.* 2009, 2010), and increasing sizes of imputation reference panels (McCarthy *et al.* 2016) now allow assay of nearly all common human genetic variants. Development of tools for meta-analysis (Willer *et al.* 2010; Winkler *et al.* 2014) has facilitated the aggregation of results across GWAS and contributed to the increasing sample sizes of genetic studies. With this changing landscape, it is worthwhile to revisit (Panagiotou and Ioannidis 2012) the common variant genome-wide threshold of *P *=* *5 × 10^−8^ considering the knowledge and data acquired in the last decade.

Instead of controlling the FWER, an inherently conservative metric, an alternative approach to multiple testing corrections is to use adjusted *P*-values to control the false discovery rate (FDR) or to use posterior probabilities to control the Bayesian FDR (Efron *et al.* 2001). Although using the Benjamini–Hochberg (B–H) procedure (Benjamini and Hochberg 1995) is the standard practice in expression quantitative trait locus (eQTL) studies and several Bayesian counterparts has also been proposed (Tang *et al.* 2007; Bogdan *et al.* 2008; Wen 2017), FDR-controlling procedures have not been widely used in GWAS. In the case of B–H, this may be due to concerns about excess estimates of FDR under the LD structure observed in genetic data (Schwartzman and Lin 2011). Recently, Brzyski *et al.* (2017) proposed a blocking strategy that groups tested variants into clusters based on LD before applying B–H and showed that this adapted procedure controlled the FDR at their target threshold of 5%. However, their analysis was limited to 364,590 variants in 5,402 samples and it is unclear how their procedure applies to meta-analysis of multiple studies. There is a need to evaluate this adapted B–H and the more conservative Benjamini–Yekutieli (B–Y) procedure (Benjamini and Yekutieli 2001) as well as other procedures that control the Bayesian FDR over a broad range of FDR thresholds at the current scale of common variant GWAS with larger samples and millions of variants.

Here, we use knowledge gathered from current studies to re-evaluate earlier common variant GWAS meta-analyses and assess the impact of different multiple testing procedures on true and false positive rate. Along with varying the *P*-value threshold which controls the FWER, we evaluate the B–H and B–Y procedures to control the FDR, and the Bayesian false discovery probability (BFDP) (Wakefield 2007) procedure to control the Bayesian FDR. We apply the multiple testing procedures to earlier common variant meta-analyses from the Global Lipids (GLGC) and GIANT GWAS consortia on several frequently studied traits: lipid levels, height, and body mass index (BMI). For the lipid traits which are correlated, we also consider the performance of the multiple testing procedures in multivariate analysis of multiple traits and compare it with multiple univariate analyses. Since the true set of causal variants for each trait is unknown, we use the latest and largest meta-analyses for each trait as the approximate “truth” to evaluate the performance of the multiple testing procedures in our empirical datasets. We supplement this analysis with simulation studies where the truth is known. Our results demonstrate that the standard 5 × 10^−8^*P*-value threshold is the best multiple testing procedure for limiting false positives and is appropriate for modest-sized studies or for resource-intensive follow-ups such as constructing animal models where the cost of follow-up for each locus is high. In contrast, a less stringent *P*-value threshold of 5 × 10^−7^ [as first suggested by the Wellcome Trust Case Control Consortium (2007)] or the adapted B–H procedure at target FDR thresholds of 5% increases power to detect true positives in large studies and can be viable for follow-ups where the cost of including a modestly greater set of false positives is low, such as gene set enrichment, pathway analysis, or high-throughput functional follow-ups. This in-depth examination provides useful guidance to investigators who are currently conducting GWAS.

## Materials and methods

### Introduction

We first consider an additive genetic model for a single continuous trait *Y* and the genotype *G_j_* at variant j=1,…, m

(1) $$Y=X^{T}\beta +G_{j}\theta_{j}+\varepsilon_{j},$$

where *X* is a *p *×* *1 vector of covariates including the intercept, *β* is a *p *×* *1 vector of covariate effects, *θ_j_* is the effect of variant *j*, and *ε_j_* is the normally distributed error with mean 0 and variance *σ^2^_j_*. This model can be applied to binary traits using a logit link function.

In a sample of *n* individuals, we wish to test the null hypotheses $H_{0,j}:\theta_{j}=0$ against the alternatives $H_{1,j}:\theta_{j}\neq 0$ for each variant *j*. Table 1 summarizes the possible outcomes for the *m* tests of which *m_0_* null hypotheses are true. For studying multiple testing procedures, we focus on the first row of the table: *R* is the total number of rejected null hypotheses, *V* the number of null hypotheses incorrectly rejected (false positives), and *S* the number of null hypotheses correctly rejected (true positives). The proportion of false positives *Q* among all rejected hypotheses is then equal to *V*/*R* for *R *>* *0 and set to 0 for *R *=* *0.

**Table 1:** Outcomes for testing multiple hypotheses

		True hypothesis		Total
		H_0_	H_1_	
Test	H_0_ rejected	*V*	*S*	*R*
	H_0_ not rejected	*U*	*T*	*m-R*
Total		*m_0_*	*m-m_0_*	*m*

Several procedures can be used to address the issue of controlling false positives when testing multiple hypotheses. In the remainder of this section we describe four such procedures, their extension to joint analysis of multiple traits, and application and assessment of these procedures in empirical and simulation studies in the context of common variant GWAS.

### FWER control

The standard procedure to correct for multiple testing in GWAS is to control the FWER, the probability of rejecting at least one true null hypothesis:

$\mathrm{FWER}=P\left(V>0\right)=P\left(Q>0\right).$

Fixed *P*-value thresholds often control the FWER by using the Bonferroni procedure which provides control of FWER at level *α* by rejecting any null hypothesis $H_{0,j}$ for variant j=1,…, m with *P*-value:

$p_{j}\leq \frac{\alpha }{m}.$

The Bonferroni criterion is conservative in two senses. One, by definition it tries to protect against making at least one mistake/false positive under the global null. The second is regarding the conservative behavior, with FWER often falling far below the desired nominal level due to correlated test statistics. This leads to loss of power. When the variants are in LD and the corresponding test statistics are correlated, one can increase the power of the Bonferroni procedure by adjusting for the effective number of independent tests (Altshuler and Donnelly 2005; Dudbridge and Gusnanto 2008; Pe’er *et al.* 2008) *m′* ≤ *m* that takes into account LD.

### FDR control

Although FWER procedures control the probability of incorrectly rejecting at least one true null hypothesis, FDR procedures control the expected proportion of incorrectly rejected true null hypotheses. At equal values of *α*, control of FDR is less conservative than control of FWER (Goeman and Solari 2014). In the context of Table 1,

FDR = EQ = EVR if R>0 0 if R=0.

The B–Y procedure controls the FDR at level α under any dependency structure by ordering the *P*-values for the *m* variants from smallest to largest: $p_{\left(1\right)},\ldots ,p_{\left(m\right)}$ and rejecting all null hypotheses $H_{0,j},\;j=1,\ldots ,\;k$ where *k* is the largest value for which: and

$p_{\left(k\right)}\leq \frac{k}{m}\left(\frac{\alpha }{c\left(m\right)}\right)$

$c\left(m\right)=\sum_{i\;=\;1}^{m}\frac{1}{i}.$

The B–H procedure, a commonly used FDR procedure that is valid when test statistics are positively correlated, is a special case of B–Y where *c(m)* is instead defined to be equal to 1. It requires an assumption of positive regression dependence on a subset (PRDS) among the test statistics as formally defined in Benjamini and Yekutieli (2001). In GWAS, the PRDS assumption means that a variants with a less significant *P*-value than another must also be more likely to have no effect on the trait (*i.e.* truly null). For the complete set of GWAS results, PRDS is likely to be violated due to correlation among tested variants. We detail below a modification of the B–H procedure proposed by Brzyski *et al.* (2017) that satisfies the PRDS assumption by filtering the full set of tested variants into independent variants using LD.

Applying the B–H or B–Y procedure to GWAS can be challenging because discoveries are counted in units of loci (clusters of nearby variants that are correlated due to LD) rather than by each individual variant. Thus, FDR-controlling procedures need to control for a subset of tested variants, typically the most strongly associated (lead) variant at each locus. Since FDR-control does not extend to a subset of the rejected null hypotheses (Goeman and Solari 2014), we adapt the B–H and B–Y procedures to GWAS by applying an approach proposed by Brzyski *et al.* (2017) We first cluster the *m* null hypotheses into m* < *m* loci by performing LD clumping on the *m* variants using a LD threshold of *r*^2^ > 0.1 and a maximal variant distance of 1 Mb (*e.g.*Fritsche *et al.* 2019). This procedure can be done using Swiss (https://github.com/statgen/swiss). We then form a set of m* P-values using the lead variant from each locus and apply the B–H or B–Y procedures on these m* P-values.

### Bayesian approach to multiple testing

A Bayesian approach to multiple testing involves calculating the posterior probability of the null hypotheses of no association given the data. For a single variant *j*, let the likelihood of the observed data $D=\left(Y,X,G_{j}\right)$ given the null hypothesis${\;H}_{0,j}$ be $p\left(D|H_{0,j}\right)$. Then by Bayes’ theorem, the probability of the null hypothesis given the data is:

$$P\left({\;H}_{0,j}|D\right)=\frac{p\left(D|H_{0,j}\right)P\left(H_{0,j}\right)}{p\left(D|H_{0,j}\right)P\left(H_{0,j}\right)+p\left(D|H_{1,j}\right)\left(1-P\left(H_{0,j}\right)\right)}=\frac{BF\times PO}{BF\times PO+1},$$

where $BF_{j}=p\left(D|H_{0,j}\right)/p\left(D|H_{1,j}\right)$ is the Bayes factor and $PO_{j}=P\left(H_{0,j}\right)/\left(1-P\left(H_{0,j}\right)\right)$ is the prior odds of no association. Here, we make the commonly accepted exchangeability assumption that every tested variant has the same prior probability of being associated with the trait, *i.e.* $1-P\left(H_{0,j}\right)=\pi_{1}$ and then conservatively estimate $\pi_{1}$ as the proportion of tested variants with *P *<* *5 × 10^−8^ in the observed summary statistics. This assumption can be easily relaxed, allowing for different priors among tested variants based on their functional annotations (Yang and Wang 2015).

For calculating an approximation of the posterior probability called the Bayesian false discovery probability (BFDP), Wakefield (2007) proposed using an approximate Bayes Factor (ABF) based on the maximum likelihood estimator (MLE) $\hat{\theta_{j}}$ of the variant effect $\theta_{j}$ as a succinct summary of the observed data D. Following Wakefield, we approximate the BF by $P\left({\hat{\theta }}_{j}|H_{0,j}\right)/P\left({\hat{\theta }}_{j}|H_{1,j}\right)$. Further assuming the sampling distribution of $\hat{\theta_{j}}$ is normal with mean $\theta_{j}$ and variance $V_{j}$ and that $\theta_{j}$ has a prior normal distribution with mean 0 and variance $W_{j}$, we calculate the ABF as a ratio of prior predictive densities ${\hat{\theta }}_{j}|H_{0,j}∼N(0,V_{j}$) and ${\hat{\theta }}_{j}|H_{1,j}∼N(0,V_{j}+W_{j})$ and use it to approximate the BFDP:

$$ABF_{j}=\frac{1}{\sqrt{1-r_{j}}}\exp\left[-\frac{Z_{j}^{2}}{2}r_{j}\right]\;$$

(2) $$\mathrm{BFD}P_{j}=\frac{ABF_{j}\times PO}{ABF_{j}\times PO+1},$$

where *Z_j_* is the test statistic and $r_{j}=W_{j}/(V_{j}+W_{j})$ is the ratio of the prior variance to the total variance where the prior variance $W_{j}$ is specified based on the study (Wakefield 2007) and $V_{j}$ is the variance of *Z_j_*. Calculating the approximate BFDP requires effect size or standard error estimates. These may not be included in publicly available GWAS results, which often are limited to *P*-values and/or *Z* statistics. If necessary, we can reliably estimate the effect size and standard error for each variant from its *Z* statistic and estimated MAF (Zhu *et al.* 2016).

Bayesian FDR is the expected proportion of false positives among all discoveries conditional on the observed data, whereas the traditional FDR is the average Bayesian FDR over many hypothetically repeated experiments (Wen 2017). Controlling the Bayesian FDR (Müller *et al.* 2004; Wen 2017) is similar to controlling the FDR except we use BFDP in the procedure in place of *P*-values. To control the Bayesian FDR in multiple hypotheses testing at level *α*, we order the BFDPs for *m* variants from smallest to largest: ${\mathrm{BFDP}}_{\left(1\right)},\ldots ,{\mathrm{BFDP}}_{\left(m\right)}$ and reject all null hypotheses $H_{0,j}$, j=1, …, k where *k* is the largest value for which:

$$\frac{\sum_{i\;=\;1}^{k}\mathrm{BFD}P_{i}}{k}\leq \alpha .$$

As with the B–H and B–Y procedures, we first cluster the tested variants into loci and then apply the BFDP procedure on the lead variant for each locus.

### Joint analysis of multiple traits

In studies of *L* correlated traits, there is potentially more power to detect association if the traits are analyzed together (Diggle *et al.* 2002). One approach is to conduct *L* parallel univariate tests and correct for testing multiple traits simultaneously (*i.e.* divide the *P*-value thresholds by *L*); an alternative is to jointly analyze the *L* traits using multivariate test statistics and then apply the usual multiple testing procedures to the *m* resulting tests.

Consider joint testing of the association between genetic variant *j* and the *L* traits under an extension of (1):

(3) $$Y_{1\times L}=X_{1\times p}^{T}\beta_{p\times L}+G_{j}\theta_{j,1\times L}+\varepsilon_{j,1\times L},$$

where *ε_j_* is normally distributed with mean $0_{1\times L\;}$and variance $\Sigma_{L\times L}$ representing the covariance matrix of the trait residuals. In (3), we test the *m* null hypotheses of no association with any trait: $H_{0,j}:\theta_{1,j}=\cdots =\theta_{L,j}=0$ for each variant j=1, …, m.

For Bonferroni and B–H/B–Y, we jointly analyzed all traits with metaMANOVA *(*Bolormaa *et al.* 2014; Ray and Boehnke 2018) using the test statistic: where *Z* is the vector of test statistics for the *L* traits, $\hat{\Omega }$ is the estimated correlation matrix for the *L* traits, and $t_{\mathrm{metaMANOVA}}$ follows an apoproximate chi-squared distribution with *L* degrees of freedom. We then apply the Bonferroni and B–Y procedures to the multivariate test statistics using the same approach as for the univariate study. To control BFDP, we use an extension (Wakefield 2007) of the ABF in (2) to multiple traits (Appendix 1 of this paper).

$t_{\mathrm{metaMANOVA}}=Z^{'}{\hat{\Omega }}^{-1}Z,$

### Empirical studies

We evaluated the performance of the multiple testing procedures in the context of common variant GWAS by using publicly available meta-analysis results from the GLGC and the GIANT consortia. For each procedure, we calculated the empirical false discovery rate (eFDR) as the number of false positive loci in the test set (*V* in Table 1) divided by the total number of significant loci identified in the test set (*R* in Table 1). Since *V* is unknown as we do not know the truth, we assume that the largest, most recent GWAS represents “truth.” We clustered variants declared significant by each procedure into loci using LD clumping. First, we ordered the significant variants by *P*-values and then using the variant with the smallest *P*-value (*i.e.* most significant) as the lead variant, we grouped all other variants that had LD threshold of *r*^2^ > 0.1 with the lead variant and within ±1 Mb of the lead variant into one locus. Next, we repeated this step on the remaining ungrouped variants until all significant variants were clustered into loci. For the adapted B–H and BFPD procedures, we first used LD clumping on all tested variants and then applied the procedures on the lead variant from each locus to obtain a set of significant loci. In the test set, we labeled loci whose lead variants had high LD (defined as *r*^2^ > 0.80) with a variant in the truth set with *P *<* *5 × 10^−8^ as true positives; the remaining loci we considered false positives. We also performed sensitivity analyses on a relaxed definition of true positive which lowered the *r*^2^ threshold to 0.60, corresponding to moderate LD between test and truth variants.

Out of four GWAS meta-analyses (Willer *et al.* 2008; Kathiresan *et al.* 2009; Teslovich *et al.* 2010; Willer *et al.* 2013) sequentially carried out for plasma high-density lipoprotein cholesterol (HDL), low-density lipoprotein cholesterol (LDL), and triglycerides (TG) levels, we picked the largest meta-analysis (Willer *et al.* 2013) with *n* = 188,577 to serve as the truth set and the second smallest meta-analysis (Kathiresan *et al.* 2009) with *n* = 19,840 to serve as the test set. We do not present results for the other two meta-analyses in the main text because one (Willer *et al.* 2008) (*n* = 8,816) had limited power and detected few significant variants and the other (Teslovich *et al.* 2010) (*n* = 100,184) had very substantial overlap in samples with the truth set so that there was insufficient sample size differences for the truth set to well approximate the truth. Of the 2,373,282 variants analyzed in both the truth and test sets, we analyzed the 2,120,069 (89%) with MAF >5% in both sets and imputed to the HapMap 3 reference panel (Altshuler *et al.* 2010).

To evaluate the multiple testing procedures over a wider range of sample sizes and genetic architectures, we also applied the procedures to meta-analyses for height and body mass index (BMI) from the GIANT consortium (Lango Allen *et al.* 2010; Speliotes *et al.* 2010; Yengo *et al.* 2018). We present results for these meta-analyses from a larger set of sequential meta-analyses (Lango Allen *et al.* 2010; Speliotes *et al.* 2010; Wood *et al.* 2014; Locke *et al.* 2015; Yengo *et al.* 2018) using the same rationale as described above for GLGC: the largest, most recent meta-analyses (Yengo *et al.* 2018) for height and BMI (*n* = 694,529 and *n* = 681,275, respectively) served as the truth sets and the smallest meta-analyses (Lango Allen *et al.* 2010; Speliotes *et al.* 2010) for each trait (*n* = 133,653 and *n* = 123,865) served as the test sets. Of the 2,282,242 variants analyzed in both meta-analyses for height, we analyzed the 2,036,404 (89%) with MAF > 5%. Of the 2,282,195 variants in both meta-analyses for BMI, we analyzed the 2,035,656 (89%) with MAF > 5% in both sets and imputed to the HapMap 3 reference panel (Altshuler *et al.* 2010).

For univariate analysis of each lipid and anthropometric trait, we used published meta-analyses results. Detailed descriptions of the statistical analyses for each of the results can be found in their respective papers (Kathiresan *et al.* 2009; Lango Allen *et al.* 2010; Speliotes *et al.* 2010; Willer *et al.* 2013; Yengo *et al.* 2018). For multivariate analysis of the three lipid traits together, we combined the univariate results using the appropriate multivariate extension for each of the procedures as described above.

### Simulation studies

To evaluate the multiple testing procedure when truth is known, we generated 1000 replicate datasets based on a simplified version of the empirical association structure observed in the latest GWAS for each of the five traits. Since we are only using the significant variants from the empirical study as causal variants in our simulation study, the number of true and false positives may differ between the two studies. We assessed the true and false positive rate of each procedure using the same method as described for our empirical studies.

To mimic the GLGC test set which consisted of European cohorts, we randomly sampled 19,840 individuals from 276,791 unrelated individuals of white British ancestry in the UK BioBank dataset. For each replicate, we used the genotypes of these individuals to generate outcomes on *n* =* *19,840 individuals for each lipid trait following model (1). We assumed the trait value *Y* is inverse normalized to maintain consistency with the empirical studies, we chose the causal variant effect sizes *θ* from the estimated values for variants with *P *<* *5 × 10^−8^ latest GLGC GWAS (the truth set), and the error term is normally distributed with mean 0 and variance equal to 1 minus the proportion of trait variance explained by the simulated causal variants. We ran association analysis with each replicate dataset using a linear regression model with no additional covariates. We took a similar approach for simulating height and BMI based on the GIANT dataset using separate generation models for the two traits. An estimated numbers of causal variants that can be detected in simulation for the five traits at different *P*-value thresholds of can be found in Supplementary Table S1.

### Data availability

Meta-analyses results for the Global Lipids Genetics Consortium are available at http://lipidgenetics.org. Meta-analyses results for the GIANT Constortium are available at https://portals.broadinstitute.org/collaboration/giant/index.php/GIANT_consortium_data_files. UK Biobank genotype data are available at http://biobank.ctsu.ox.ac.uk/crystal/label.cgi?id=263.

Supplementary material is available at figshare DOI: https://doi.org/10.25387/g3.13211507.

## Results

We applied the multiple testing procedures to HDL, LDL, TG, height, and BMI to assess their performances for different sample sizes and genetic structures.

### *P*-value threshold

Applying various fixed *P*-value thresholds to the empirical GLGC and GIANT test sets, we observed as expected that the empirical false discovery rate (eFDR) generally increased as the *P*-value threshold increased (Tables 2 and 3). The lone exception (for HDL) likely reflected statistical noise due to the small number of identified loci.

**Table 2:** Empirical and simulation results for all traits and commonly used thresholds

		Empirical			Simulation		
		Positives		eFDR^b^	Positives		eFDR (SE)
		False	True^a^		False	True	
HDL (n_test_ = 19,840 n_truth_ = 188,577)	P = 5 × 10^−8^	1	16	5.9%	0.28	9.5	2.9% (0.33%)
	P = 5 × 10^−7^	1	18	5.3%	0.89	12	6.7% (0.45%)
	BH = 5%	1	18	5.3%	0.70	12	5.6% (0.43%)
	BY = 5%	0	14	0%	0.17	8.2	2.0% (0.31%)
	BFDP = 5%	1	17	5.6%	0.41	10	4.0% (0.37%)
LDL (n_test_ = 19,840 n_truth_ = 188,577)	5 × 10^−8^	0	14	0%	0.19	13	1.5% (0.19%)
	5 × 10^−7^	3	16	16%	0.71	16	4.2% (0.27%)
	BH = 5%	3	16	16%	0.67	16	4.0% (0.27%)
	BY = 5%	0	14	0%	0.13	12	1.1% (0.17%)
	BFDP = 5%	2	17	11%	0.37	14	2.5% (0.22%)
TG (n_test_ = 19,840 n_truth_ = 188,577)	5 × 10^−8^	1	8	11%	0.11	9.0	1.2% (0.21%)
	5 × 10^−7^	2	10	17%	0.54	10	4.9% (0.37%)
	BH = 5%	2	9	18%	0.32	10	3.1% (0.30%)
	BY = 5%	0	8	0%	0.05	8.5	0.58% (0.16%)
	BFDP = 5%	1	10	9.1%	0.36	9.8	3.6% (0.32%)
Height (n_test_ = 133,653 n_truth_ = 693,529)	5 × 10^−8^	0	157	0%	1.6	181	0.89% (0.042%)
	5 × 10^−7^	1	217	0.46%	4.9	223	2.2% (0.055%)
	BH = 5%	2	351	0.57%	22	301	6.8% (0.086%)
	BY = 5%	0	197	0%	4.3	217	2.0% (0.054%)
	BFDP = 5%	2	338	0.59%	28	317	8.1% (0.077%)
BMI (n_test_ = 123,865 n_truth_ = 681,275)	5 × 10^−8^	0	22	0%	0.62	39	1.6% (0.13%)
	5 × 10^−7^	0	37	0%	2.7	58	4.4% (0.19%)
	BH = 5%	1	41	2.4%	6.6	77	7.9% (0.19%)
	BY = 5%	0	20	0%	0.83	41	2.0% (0.15%)
	BFDP = 5%	0	35	0%	3.9	67	5.4% (0.18%)

a Number of loci in truth set for HDL: 89, LDL: 72, TG: 60, height: 1100, BMI: 724.

b eFDR is calculated as number of false positives divided by sum of true and false positives.

**Table 3:** Empirical and simulation results for *P*-value thresholds

Trait	Threshold (*P*-value)	Empirical				Simulation			
		Positives		eFDR^b^	Δ in #of sig. loci (% true)	Positives		eFDR	Δ in #of sig. loci (% true)
		False	True^a^			False	True		
HDL (n_test_ = 19,840 n_truth_ = 188,577)	5 × 10^−8^	1	16	5.9%	–	0.28	9.5	2.9%	–
	5 × 10^−7^	1	18	5.3%	+2 (100%)	0.89	12	6.7%	+3.6 (83%)
	5 × 10^−6^	8	21	28%	+10 (30%)	4.2	17	20%	+8.2 (60%)
LDL (n_test_ = 19,840 n_truth_ = 188,577)	5 × 10^−8^	0	14	0%	–	0.19	13	1.5%	–
	5 × 10^−7^	3	16	16%	+5 (40%)	0.71	16	4.2%	+3.9 (87%)
	5 × 10^−6^	10	19	34%	+10 (30%)	4.6	22	17%	+9.1 (58%)
TG (n_test_ = 19,840 n_truth_ = 188,577)	5 × 10^−8^	1	8	11%	–	0.11	9.0	1.2%	–
	5 × 10^−7^	2	10	17%	+3 (67%)	0.54	10	4.9%	+1.9 (77%)
	5 × 10^−6^	6	15	29%	+9 (56%)	3.8	13	22%	+6.1 (47%)
Height (n_test_ = 133,653 n_truth_ = 693,529)	5 × 10^−8^	0	157	0%	–	1.6	181	0.89%	–
	5 × 10^−7^	1	217	0.46%	+61 (98%)	4.9	223	2.2%	+46 (93%)
	5 × 10^−6^	2	312	0.64%	+96 (99%)	16	283	5.4%	+72 (84%)
BMI (n_test_ = 123,865 n_truth_ = 681,275)	5 × 10^−8^	0	22	0%	–	0.62	39	1.6%	–
	5 × 10^−7^	0	37	0%	+15 (100%)	2.7	58	4.4%	+22 (90%)
	5 × 10^−6^	1	55	1.8%	+19 (95%)	11	90	11%	+41 (79%)

a Number of loci in truth set for HDL: 89, LDL: 72, TG: 60, height: 1100, BMI: 724.

b eFDR is calculated as number of false positives divided by sum of true and false positives.

**Figure 1:**
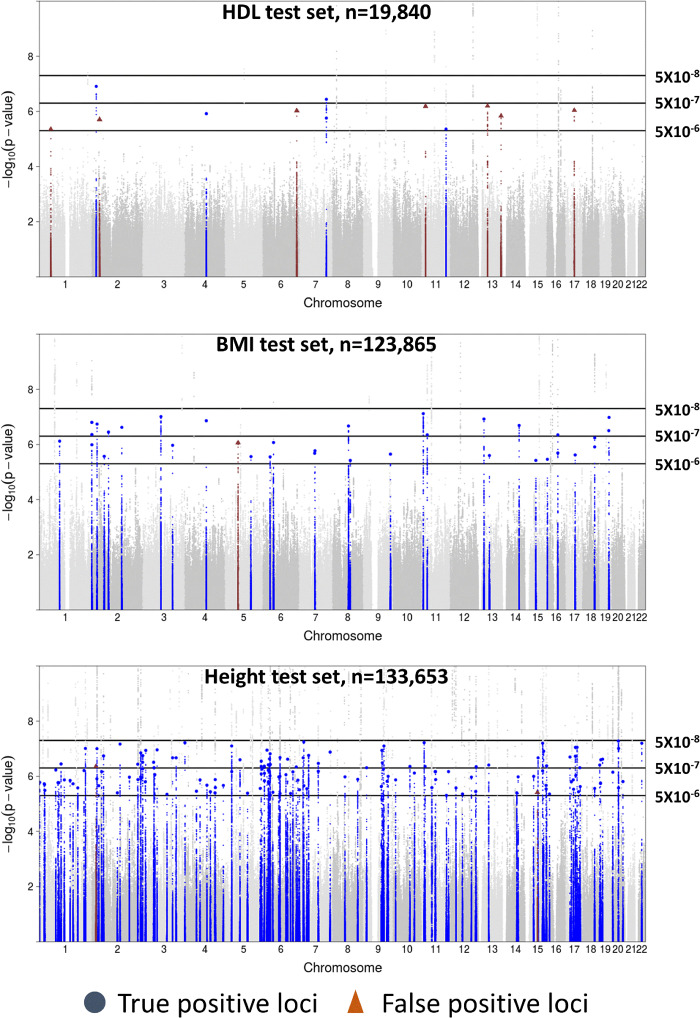
Manhattan plot of empirical *P*-value thresholds for variants with *P* ≥ 5x10^−8^. Plots of different *P*-value thresholds applied to empirical test sets for HDL, BMI, and height. Colored variants depict true positive loci (blue) and false positive loci (red) for variants with *P* ≥ 5x10^−8^. Lead variants for true and false positive loci are represented by large blue circles and large triangles, respectively.

For height and BMI, we identified substantially more loci by relaxing the threshold from *P *=* *5 × 10^−8^ to *P *=* *5 × 10^−7^ with nearly all these new loci being true positives (Table 2 and Figure 1): 60 of 61 (98%) for height; 15 of 15 (100%) for BMI (Table 3). Further relaxing the threshold from *P *=* *5 × 10^−7^ to *P *=* *5 × 10^−6^ maintained high proportions of true positives among the additional loci: 95 of 96 (99%) for height, 18 of 19 (95%) for BMI. For the lipid traits in the GLGC test set, relaxing the threshold from *P *=* *5 × 10^−8^ to *P *=* *5 × 10^−7^ resulted in HDL, LDL, and TG gaining 2, 5, and 3 loci with 2, 2, and 2 (100%, 40%, and 67%) being true positives. Further relaxing the threshold from *P *=* *5 × 10^−7^ to *P *=* *5 × 10^−6^ resulted in ≤ 56% of the additional loci being true positives for the lipid traits.

We observed in the GLGC- and GIANT-based simulated datasets that the average eFDR increased as the *P*-value threshold increased for all traits (Table 3); the inconsistency described before for the empirical HDL test set disappeared when we averaged over 1,000 simulation replicates. Consistent with the empirical results, there was a clear difference in the proportion of true positives between the lipid and anthropometric traits in the simulated results (Table 3). Relaxing the threshold from *P *=* *5 × 10^−8^ to *P *=* *5 × 10^−7^ in the simulated datasets resulted in an average of 77% to 87% of the additional loci being true positives for the lipid traits and 93% and 90% for height and BMI. Further relaxing the threshold from *P *=* *5 × 10^−7^ to *P *=* *5 × 10^−6^ resulted in 47% to 60% of the additional loci being true positives for lipids, and 84% and 79% for height and BMI.

To address whether the higher rates of true positives we observed when relaxing the *P*-value threshold for height and BMI compared to those for lipids were the result of differences in sample sizes, we simulated test sets for height and BMI at the same sample sizes (*n* = 8,816 and *n* = 19,840) as the GLGC meta-analyses. For both traits, an increase in sample size generally led to higher proportion of true positives gained from relaxing the *P*-value threshold (Table 4), suggesting a better yield of true positives by using relaxed thresholds in larger samples than in smaller ones.

**Table 4:** Effect of sample size on simulation results for *P*-value thresholds

Trait	Threshold (*P*-value)	*n* = 8,816				*n* = 19,840				*n* = 133,653 or 123,865			
		Positives		eFDR^b^	Δ sig. loci (% True positive)	Positives		eFDR	Δ sig. loci (% True positive)	Positives		eFDR	Δ sig. loci (% True positive)
		False	True^a^			False	True			False	True		
Height	5 × 10^-8^	0.04	0.90	4.3%	–	0.03	11	0.27%	–	1.6	181	0.89%	–
	5 × 10^−7^	0.40	2.2	15%	+1.7 (78%)	0.32	18	1.7%	+7.4 (96%)	4.9	223	2.2%	+46 (93%)
	5 × 10^−6^	3.0	5.9	34%	+6.3 (58%)	3.6	30	11%	+15 (79%)	16	283	5.4%	+72 (84%)
BMI	5 × 10^−8^	0.04	0.20	17%	–	0.09	1.5	5.7%	–	0.62	39	1.6%	–
	5 × 10^−7^	0.34	0.41	45%	+0.51 (41%)	0.46	2.4	16%	+1.3 (72%)	2.7	58	4.4%	+22 (90%)
	5 × 10^−6^	3.1	1.2	73%	+3.5 (21%)	3.2	4.6	41%	+5.0 (44%)	11	90	11%	+41 (79%)

a Number of loci in truth set for HDL: 89, LDL: 72, TG: 60, height: 1100, BMI: 724.

b eFDR is calculated as number of false positives divided by sum of true and false positives.

### Benjamini–Hochberg and Benjamini–Yekutieli procedures

As expected, empirical results for the two FDR controlling procedures showed B–Y was conservative, resulting in eFDR far below the target FDR threshold for all traits at commonly used (5-15%; Table 5) and more extreme (1–25%; Supplementary Table S2) thresholds. B–H controlled the eFDR at the target thresholds (Table 6 and Supplementary Table S3) for height and BMI but not lipid traits, likely because the number of lipid trait discoveries was modest (≤ 26 loci for B–H) so that even a small change in numbers of true and false positives substantially influenced estimated eFDR. When the total number of discoveries is small, it is more useful to assess control of FDR averaged over a large number of datasets in simulation since the variance of the eFDR based on a single empirical study can be quite large.

**Table 5:** Empirical and simulation results for Benjamini–Yekutieli procedure

Trait	Threshold (FDR)	Empirical				Simulation		
		Positives		eFDR^b^		Positives		eFDR
		False	True^a^			False	True	
HDL (n_test_ = 19,840 n_truth_ = 188,577)	5%	0	14	0%		0.17	8.2	2.0%
	10%	1	16	5.9%		0.25	9.1	2.7%
	15%	1	16	5.9%		0.31	9.6	3.1%
LDL (n_test_ = 19,840 n_truth_ = 188,577)	5%	0	14	0%		0.13	12	1.1%
	10%	0	14	0%		0.19	13	1.5%
	15%	0	15	0%		0.26	13	1.9%
TG (n_test_ = 19,840 n_truth_ = 188,577)	5%	0	8	0%		0.05	8.5	0.58%
	10%	0	8	0%		0.06	8.7	0.68%
	15%	1	8	11%		0.11	9.0	1.2%
Height (n_test_ = 133,653 n_truth_ = 693,529)	5%	0	197	0%		4.3	217	2.0%
	10%	1	234	0.43%		6.3	235	2.6%
	15%	1	249	0.40%		7.9	246	3.1%
BMI (n_test_ = 123,865 n_truth_ = 681,275)	5%	0	20	0%		0.83	41	2.0%
	10%	0	22	0%		1.4	47	2.9%
	15%	0	26	0%		1.8	52	3.4%

a Number of loci in truth set for HDL: 89, LDL: 72, TG: 60, height: 1100, BMI: 724.

b eFDR is calculated as number of false positives divided by sum of true and false positives.

**Table 6:** Empirical and simulation results for Benjamini–Hochberg procedure

Trait	Threshold (FDR)	Empirical			Simulation		
		Positives		eFDR^b^	Positives		eFDR
		False	True^a^		False	True	
HDL (n_test_ = 19,840 n_truth_ = 188,577)	5%	1	18	5.3%	0.70	12	5.6%
	10%	5	18	22%	1.3	13	8.5%
	15%	6	20	23%	1.7	14	11%
LDL (n_test_ = 19,840 n_truth_ = 188,577)	5%	3	16	16%	0.67	16	4.0%
	10%	4	16	20%	1.1	18	6.0%
	15%	7	17	29%	1.7	18	8.6%
TG (n_test_ = 19,840 n_truth_ = 188,577)	5%	2	9	18%	0.32	10	3.1%
	10%	5	10	33%	0.63	11	5.6%
	15%	5	10	33%	0.96	11	8.0%
Height (n_test_ = 133,653 n_truth_ = 693,529)	5%	2	351	0.57%	22	301	6.8%
	10%	4	421	0.94%	37	331	10%
	15%	8	468	1.7%	50	351	13%
BMI (n_test_ = 123,865 n_truth_ = 681,275)	5%	1	41	2.4%	6.6	77	7.9%
	10%	1	47	2.1%	11	91	11%
	15%	1	55	1.8%	16	102	14%

a Number of loci in truth set for HDL: 89, LDL: 72, TG: 60, height: 1100, BMI: 724.

b eFDR is calculated as number of false positives divided by sum of true and false positives.

Simulation results for B–Y were consistent with empirical results in showing that B–Y is overly conservative for all five traits and all target FDR thresholds (Table 5 and Supplementary Table S2). For example, the observed eFDR for target threshold of 15% is < 3.4% for all traits and the equivalent *P*-value threshold for that target threshold is 10 times more stringent for B–Y than B–H (Supplementary Table S4). Compared to the empirical results, B–H did a better job of controlling eFDR at the commonly used thresholds (Table 6) for all traits; only for height at 5% and BMI at 5% did B–H show noticeable excess in eFDR (6.8% for height, 7.9% for BMI). When we relaxed our criterion for defining a true positive (see below), excess eFDR for height and BMI decreased (eFDR = 5.5% and 5.2%) (Supplementary Table S5). eFDR was well-controlled at high thresholds 20% and 25% for all five traits but poorly-controlled at low thresholds 1% and 3% (Supplementary Table S3).

We investigated whether FDR control for B–H and B–Y extended across sample sizes by using simulated datasets for height and BMI at *n* = 8,816, *n* = 19,840 and *n* = 133,653 (height) or 123,865 (BMI). Both procedures controlled eFDR at the target FDR thresholds 5-15% for height (Supplementary Tables S6 and S7); BMI showed excess eFDR under B–H for all test sets which disappeared under the relaxed definition of true positives (Supplementary Table S5; data not shown for smaller sample sizes).

### Bayesian false discovery probability

For the BFDP procedure, we estimated the prior probability of association at a variant site ($\pi_{1}$) separately for each test set using the proportion of tested variants with *P *<* *5 × 10^−8^. Empirical results showed that eFDR was well controlled for height and BMI at target Bayesian FDR thresholds 1-25% but poorly controlled for lipid traits (Table 7 and Supplementary Table S8), again likely due to the smaller number of discoveries for lipid traits (≤ 24 loci for BFDP).

**Table 7:** Empirical and simulation results for BFDP procedure

Trait	Threshold (Bayesian FDR)	Empirical						Simulation			
		$\hat{\pi_{1}}$ ^a^	Positives		eFDR^c^	$\hat{\pi_{1}}$ ^d^	Positives			eFDR
			False	True^b^			False		True	
HDL (n_test_ = 19,840 n_truth_ = 188,577)	5%	1.3 × 10^−4^	1	17	5.6%	8.7 × 10^−5^	0.41		10	4.0%
	10%		4	17	19%		0.76		12	6.1%
	15%		6	18	25%		1.2		13	8.4%
LDL (n_test_ = 19,840 n_truth_ = 188,577)	5%	1.3 × 10^−4^	2	17	11%	9.6 × 10^−5^	0.37		14	2.5%
	10%		5	17	23%		0.83		16	4.9%
	15%		6	18	25%		1.3		18	7.0%
TG (n_test_ = 19,840 n_truth_ = 188,577)	5%	2.1 × 10^−4^	1	10	9.1%	1.6 × 10^−4^	0.36		9.8	3.6%
	10%		4	10	29%		1.0		11	8.4%
	15%		4	12	25%		1.6		12	12%
Height (n_test_ = 133,653 n_truth_ = 693,529)	5%	2.0 × 10^−3^	2	338	0.59%	2.9 × 10^−3^	28		317	8.1%
	10%		7	406	1.7%		51		356	13%
	15%		9	468	1.9%		76		385	17%
BMI (n_test_ = 123,865 n_truth_ = 681,275)	5%	3.6 × 10^−4^	0	35	0%	5.2 × 10^−4^	3.9		67	5.4%
	10%		0	43	0%		7.2		82	8.0%
	15%		0	50	0%		11		93	10%

a $\hat{\pi_{1}}$ is the estimated prior probability of association at a variant site equal to the proportion of tested variants with *P* < 5 × 10^−8^.

b Number of loci in truth set for HDL: 89, LDL: 72, TG: 60, height: 1100, BMI: 724.

c eFDR is calculated as number of false positives divided by sum of true and false positives.

d Average $\hat{\pi_{1}}$ in 1,000 replicate datasets.

Simulation results for BFDP showed that eFDR was generally well controlled at target Bayesian FDR thresholds 5-15% (Table 7) for all traits except height (eFDR = 8.1%, 13%, and 17%). For more extreme thresholds (Supplementary Table S8), eFDR was controlled at 1 and 3% for lipid traits, albeit with excess eFDR for HDL at 1%; eFDR was controlled at 20% and 25% for all traits.

### Multi-trait analysis results for lipids

In empirical results (Supplementary Table S9), the *P *=* *5 × 10^−8^ threshold had the lowest eFDR for the parallel univariate tests both adjusted (Bonferroni corrected threshold of 1.67 × 10^−8^) and unadjusted (5 × 10^−8^) for testing three traits. For the multivariate tests, the *P *=* *5 × 10^−8^ and *P *=* *5 × 10^−7^ thresholds had identical eFDR of 0%. Between the three sets of thresholds, the multivariate analysis had the lowest eFDR as well as the highest proportion of true positive discoveries when relaxing the *P*-value thresholds. For both the B–H and BFDP procedures, multivariate tests had lower eFDR than the univariate tests but only the multivariate B–H procedure controlled the eFDR at target thresholds 5-15%.

In simulation results (Supplementary Table S10), the *P *=* *5 × 10^−8^ threshold had the lowest eFDR for all three sets of tests. Consistent with empirical results, multivariate tests had the lowest eFDR at all three *P*-value thresholds and better true positive rate for relaxing thresholds compared with the univariate tests. For the B–H and BFDP procedure, both univariate and multivariate tests controlled the eFDR at target thresholds 5-15%.

### Sensitivity analyses

We defined true positives in the test set strictly as loci whose lead variants had LD threshold of r^2^ > 0.80 with a genome-wide significant (*P* < 5 × 10^−8^) variant in the truth set. We chose this strict criterion to avoid underestimating the number of false positives in our analysis but it likely led to overestimation of eFDR. To assess the impact of this, we repeated our simulation analyses using a relaxed definition of true positives by lowering the LD threshold requirement to 0.60 and the *P*-value requirement to 5 × 10^−7^ (Supplementary Table S5). As expected, under this relaxed definition we observed fewer false positives and occurrences of excess eFDR largely disappeared as well. For example, simulation results for height using the BFDP procedure at FDR thresholds of 10% and 15% showed eFDR of 13% and 17% under the strict definition and 10% and 14% under the relaxed definition.

In addition to our LD-based definitions, we used physical distance to define loci and true positives. We grouped variants within ±1Mb of the lead variants into loci and defined true positives as loci whose lead variants were within ±50kb of a genome-wide significant variant in the truth set. The analyses results (Supplementary Table S11) showed that the distance-based definitions led to smaller numbers of true and false positives for all traits and multiple testing procedures.

## Discussion

In this paper, we leverage the sequentially growing nature of GWAS meta-analyses to evaluate true and false positive rate of *P*-value thresholds and other multiple testing procedures. Although the standard procedure for identifying significant associations in common variant GWAS is to use a *P*-value threshold of 5 × 10^−8^, relaxing the significance criteria, whether through use of less stringent *P*-value thresholds or controlling for alternative error rate measures such as FDR (depending on the target threshold) increases the number of identified loci. We demonstrated that a substantial proportion of the additional loci identified by relaxed *P*-value thresholds are true positives, with larger proportions of true positives in analysis of larger samples.

### Application to downstream analyses

GWAS identify trait-associated variants and loci based on association analysis of millions of variants. The identified loci are often further validated in replication studies before being used for statistical and functional analyses to identify causal genes, variants, and mechanisms. Although relaxed *P*-value thresholds are often used to generate the list of loci for replication, the expected true and false discovery rates under different thresholds have not been quantified. We showed by simulation for common variant GWAS with sample size > 100,000 that 90-93% of additional discoveries with *P*-values between 5 × 10^−8^ and 5 × 10^−7^ were true positives, representing true associations that would be lost under a more stringent threshold. However, for more modest sample sizes (∼20,000), our simulation showed that only 77-87% of additionally discovered loci with *P*-values between 5 × 10^−8^ and 5 × 10^−7^ were true positives. Here, investigators should exercise caution when relaxing the significance threshold for replication studies as the increase to replicated associations may not outweigh the excess false discovery rate.

For follow-up studies such as constructing animal models where the per-locus cost of follow-up is high, a stringent *P*-value threshold of 5 × 10^−8^ is appropriate in both large and modest-sized studies to generate a highly accurate list of associated loci. However, such threshold may be unhelpfully conservative for analyses where including (many) more true loci at the cost of (a few) more false positives is acceptable such as gene-set enrichment or pathway analysis. In these situations, a relaxed threshold of 5 × 10^−7^ may be better served to prioritize GWAS results for downstream analyses.

### FDR- and Bayesian FDR-control

FDR-control is an appropriate choice for practitioners who are willing to tolerate some proportion of false positive discoveries as long as it can be controlled below a target threshold. At equal thresholds, controlling the FDR is less conservative than controlling the FWER and thus expands the GWAS-identified set of associated loci for downstream analysis, especially for highly polygenic traits. We showed that the B–H procedure adapted for GWAS (see *Materials and Methods*) provided approximate control of the empirical estimate of FDR (eFDR) for the tested traits and samples at target thresholds 5-25%. The B–Y procedure is far too conservative in GWAS as the correction factor which removes assumptions on the dependency structure of test statistics is unnecessary under the adapted B–H procedure which forms independent test statistics using the lead variants from each locus.

For BFDP, a Bayesian alternative to B–H, we estimated the proportion of trait-associated variants $\pi_{1}$ using the proportion of tested variants with *P*-values less than 5 × 10^−8^ and found the Bayesian FDR to be reasonably well controlled at thresholds of 5-25%. For comparison, when we estimated $\pi_{1}$ as the number of loci with lead variant *P *<* *5 × 10^−8^ divided by 1 million [an estimate for the total number of independent common variants in the genome (Altshuler and Donnelly 2005; Pe’er *et al.* 2008)], the resulting lower $\pi_{1}$ estimates led to conservative results (Supplementary Table S12).

#### Comparison between procedures

A *P*-value threshold has the advantages of familiarity, simplicity, and ease of implementation, whereas B–H and BFDP control the eFDR across a range of sample sizes. A stringent *P*-value threshold is needed if our primary goal is to limit the number of false positives as both B–H and BFDP struggled to control the eFDR at low target thresholds 1% and 3%. In addition, the *P*-value threshold can also be used to control the Per Family Error Rate (PFER) as discussed in Gordon *et al.* (2007) since the Bonferroni procedure can be trivially extended to control PFER.

#### Limitations

Our analysis is based on five anthropometric and lipid traits which obviously do not fully reflect the wide range of phenotypes studied in GWAS. However, our simulated traits do represent different levels of polygenicity ranging from HDL with 89 causal variants and some larger effect sizes (Supplementary Figure S1) to height with 1100 causal variants and generally smaller effect sizes, covering a wide range of genetic architectures for quantitative phenotypes.

Our analysis is also limited to studies of European-origin individuals. Further work using test and truth sets with non-European samples would be welcome to confirm that our findings are applicable more generally.

We relied on publicly available meta-analyses results from the GLGC and GIANT consortia for our empirical and simulation studies. However, imputation qualities were not provided for these datasets, which raises the concern that our analyses would have missed the effects of poorly imputed variants. Since we restricted our analyses to ∼2 million HapMap3 common variants with consistently high imputation r-squared across past studies, we believe our results were not substantially affected by variation in imputation quality.

We defined true and false positive discoveries in our empirical analysis using the largest, most recent common variant GWAS which we called the truth sets. Since our empirical test set came from the same sequential set of meta-analyses as the truth set, the samples in our test set were a complete subset of those in the truth set. Although this procedure did not guarantee the (unknown) list of loci truly associated with each tested trait, the truth sets served as reasonable approximations when there were considerable sample size differences between the truth and test sets. When we repeated our analysis for smaller test sets (*n* = 8,816), we observed well-controlled eFDR for the B–H procedure as expected. However, in larger test sets (*n* = 253,288), we observed noticeable excess in eFDR which disappeared in simulations. This is likely because the sample size differences between the larger empirical test sets and the approximate truth set is small enough that the “truths” may no longer be accurate. When this occurs, false positive discovery in the test set can become biased since a false positive in the test set may be discovered (and thus become a true positive) in a subsequent study larger than the truth set. In simulated datasets where the truth was known, the eFDR was well-controlled.

Association results on the same samples may differ owing to changes in quality control and/or statistical analysis. For example, some studies may choose not to apply genomic control which can then lead to inflated *P*-values due to confounding from population stratification. In this scenario, relaxing the significance threshold may not be appropriate since the number of false positives would already be higher than expected. We did not observe substantial genomic inflation in our analyses (average lambda GC of 1.04, 95% CI: 1.02, 1.05) and so could not easily investigate how our results would have changed depending on whether GC is or is not applied.

Although our results showed only modest differences between multivariate and univariate tests in terms of multiple testing corrections, we only considered the case of three correlated traits as opposed to the tens to thousands of traits that might simultaneously be tested in metabolomics or imaging studies. With many traits, combining univariate tests but not adjusting for multiple traits will surely lead to excess false positive discoveries (as even our limited results demonstrated), whereas adjusting for hundreds of traits may be overly conservative. Here, multivariate tests may represent an attractive option.

In this study, we focused on common variants (MAF > 5%). This allowed us to construct likely highly accurate truth sets of loci based on serial common variant GWAS for anthropometric and lipid traits to facilitate the evaluation of different procedures for multiple testing corrections. We plan next to consider multiple testing in the context of rare variant analysis (Pulit *et al*. 2017; Lin 2019) which necessarily will increase the number of hypothesis tests for which we need to correct and introduce the consideration of gene- and more generally set-based association tests.

#### Summary

In this study, we evaluated the performance of four procedures for multiple testing corrections in the context of common variant GWAS: *P*-value thresholds, B–H and B–Y for FDR control, and BFDP for Bayesian FDR control. We have shown that for studies based on large samples, using a less stringent *P*-value threshold of 5 × 10^−7^ or use of FDR-controlling procedure (B–H) at target threshold of 5% substantially increases the number of true positive discoveries that can be used in downstream analyses while only modestly increasing false positives compared with the commonly used 5 × 10^−8^*P*-value threshold. The latter threshold remains the preferred choice for modest-sized studies or when a stringently curated list of loci is desired. Finally, we show that FDR-control largely extends across sample sizes with a few exceptions that disappear under a relaxed definition of true positives and FDR-controlling procedures can be similarly applied to large and modest-sized studies.

## Appendix 1: Multivariate BFDP

Consider joint testing of the association between a genetic variant and *L* traits under model (3). To match our analysis, we set *L *=* *3 for the rest of this section but this multivariate extension can be applied to any number of traits.

As in the univariate case described in *Materials and Methods* and in Wakefield (2007), we approximate the multivariate Bayes’ factor by $P\left(\hat{\theta }|H_{0}\right)/P\left(\hat{\theta }|H_{1}\right)$ where $\hat{\theta }=({\hat{\theta }}_{1},{\hat{\theta }}_{2},{\hat{\theta }}_{3})$ is the estimated vector of variant effect sizes for the three traits. We assume the sampling distribution of $\hat{\theta }$ is multivariate normal with mean θ and variance V and that θ has a prior multivariate normal distribution with mean 0 and variance W. If ρ is the 3 × 3 matrix of correlation between the traits and $\rho_{ij}$ is the correlation between traits *i* and *j*, then:

$$V=\left[\begin{array}{ccc} V_{1} & \rho_{12}\sqrt{V_{1}V_{2}} & \rho_{13}\sqrt{V_{1}V_{3}}\\ \rho_{21}\sqrt{V_{2}V_{1}} & V_{2} & \rho_{23}\sqrt{V_{2}V_{3}}\\ \rho_{31}\sqrt{V_{3}V_{1}} & \rho_{32}\sqrt{V_{3}V_{2}} & V_{3} \end{array}\right]$$

$$W=\left[\begin{array}{ccc} W_{1} & \rho_{12}\sqrt{W_{1}W_{2}} & \rho_{13}\sqrt{W_{1}W_{3}}\\ \rho_{21}\sqrt{W_{2}W_{1}} & W_{2} & \rho_{23}\sqrt{W_{2}W_{3}}\\ \rho_{31}\sqrt{W_{3}W_{1}} & \rho_{32}\sqrt{W_{3}W_{3}} & W_{3} \end{array}\right],$$

where $V_{k}$ is the sample variance and $W_{k}$ is the prior variance for trait *k*. Finally, the multivariate approximate Bayes’ factor (ABF) can be calculated as a ratio of prior predictive densities $\hat{\theta }|H_{0}∼\mathrm{MVN}(0,\;V$) and $\hat{\theta }|H_{1}∼\mathrm{MVN}(0,V+W)$ and used to approximate the BFDP:

$$ABF_{\mathrm{multi}}={\left|V\right|}^{-\frac{1}{2}}{\left|V+W\right|}^{\frac{1}{2}}\exp\left[\frac{{\hat{\theta }}^{T}\left(-V^{-1}+{\left(V+W\right)}^{-1}\right)\hat{\theta }}{2}\right]$$

$$\mathrm{BFD}P_{\mathrm{multi}}=\frac{ABF_{\mathrm{multi}}\times PO}{ABF_{\mathrm{multi}}\times PO+1}.$$

For the prior odds of no association, we estimate the prior probability of being associated with the three traits (${\hat{\pi_{1}}}_{\mathrm{multi}}$) as the average of the $\hat{\pi_{1}}$’s from each trait which is calculated as described in *Materials and Methods*.

To obtain the combined univariate results for the multiple testing procedures, we merged the dataset for the three lipid traits and calculated the number of unique variants that were deemed significant by the multiple testing methods. Then, we used the procedure described in *Materials and Methods* to determine which of the significant variants were true or false positives.
